# High-Density Genetic Map Construction and Quantitative Trait Locus Analysis of Fruit- and Oil-Related Traits in *Camellia oleifera* Based on Double Digest Restriction Site-Associated DNA Sequencing

**DOI:** 10.3390/ijms25168840

**Published:** 2024-08-14

**Authors:** Ping Lin, Jingyu Chai, Anni Wang, Huiqi Zhong, Kailiang Wang

**Affiliations:** 1 State Key Laboratory of Tree Genetics and Breeding, Research Institute of Subtropical Forestry, Chinese Academy of Forestry, Hangzhou 311400, China; linping80@126.com (P.L.); c0808jy@163.com (J.C.); wanganni926@126.com (A.W.); zhonghuiqi2023@163.com (H.Z.); 2 Zhejiang Key Laboratory of Forest Genetics and Breeding, Research Institute of Subtropical Forestry, Chinese Academy of Forestry, Hangzhou 311400, China

**Keywords:** *Camellia oleifera*, double digest restriction site-associated DNA sequencing (ddRAD-Seq), genetic map, QTLs, fruit- and oil-related traits

## Abstract

*Camellia oleifera*, an important tree species and source of edible oil in China, has received significant attention owing to the oil’s high unsaturated fatty acid content, which has benefits for human health. However, the mechanisms underlying *C. oleifera* yield and oil quality are largely unknown. In this study, 180 F_1_ progenies were obtained from two parents with obvious differences in fruit- and oil-related traits. We constructed a high-density genetic map using a double digest restriction site-associated DNA sequencing (ddRAD-Seq) strategy in *C. oleifera*. This map spanned 3327 cM and anchored 2780 markers in 15 linkage groups (LGs), with an average marker interval of 1.20 cM. A total of 221 quantitative trait loci (QTLs) associated with fruit- and oil-related traits were identified across three years’ worth of phenotypic data. Nine QTLs were detected simultaneously in at least two different years, located on LG02, LG04, LG05, LG06, and LG11, and explained 8.5–16.6% of the phenotypic variation in the corresponding traits, respectively. Seventeen major QTLs were obtained that explained 13.0–16.6% of the phenotypic variance. Eleven and five flanking SNPs of major QTLs for fruit- and oil-related traits were detected which could be used for marker-assisted selection in *C. oleifera* breeding programs. Furthermore, 202 potential candidate genes in QTL regions were identified based on the collinearity of the genetic map and the *C. oleifera* “CON” genome. A potential regulatory network controlling fruit development and oil biosynthesis was constructed to dissect the complex mechanism of oil accumulation. The dissection of these QTLs will facilitate the gene cloning underlying lipid synthesis and increase our understanding in order to enhance *C. oleifera* oil yield and quality.

## 1. Introduction

*Camellia oleifera*, a species belonging to the genus *Camellia* (Theaceae), originates from China and has a cultivation history of over 2000 years in south China [1]. It is widely recognized as a significant source of edible oil in China, with a cultivated area exceeding 4 million hectares [2,3]. It is also widely used in cosmetics, ink, lubricants, etc. [4,5]. The oil extracted from the seeds of *C. oleifera*, known as Camellia oil, exhibits a distinctive chemical composition and possesses notable medicinal and healthcare properties. It is rich in unsaturated fatty acids (comprising over 90% of the total oil), of which the monounsaturated fatty acid, oleic acid, accounts for about 80% of the total oil content [6]. Camellia oil also contains squalene, tea polyphenol, and other bioactive substances [7] which are beneficial for reducing the level of blood cholesterol and treating cardiovascular and cerebrovascular diseases [7,8]. For these reasons, *C. oleifera* has received a significant amount of attention, and the yield and quality of the seed oil have been continuously selected as the primary targets in *C. oleifera* breeding [1]. 

Due to the long breeding cycle, *C. oleifera* poses unique challenges for genetic improvement, and there is an urgent need to improve upon traditional breeding methods [9]. High-resolution genetic maps are indispensable to many genetic studies and molecular breeding programs, such as molecular-marker-assisted selection, fine-scale quantitative trait locus (QTL) mapping, genome scaffolding and assembly, and comparative genomic analysis. Advances in the genetic map construction and QTL analysis of *Brassica napus*, soybean [10,11,12], oil palm [13], and other plants have revealed novel findings in fruit development and the oil biosynthesis pathway and provided valuable genomic resources for genetic improvement in oil crops [14,15,16,17]. The application of molecular-marker-assisted breeding is anticipated to expedite *C. oleifera* breeding cycles. However, the complexity of *C. oleifera*’s genetic background, a scarcity of molecular markers, and the challenges in hybrid population creation have hindered the progress of genetic map construction and molecular-marker-assisted selection studies. *C. oleifera* is a self-incompatible tree with a highly heterozygous genome (heterozygosity 2.52%), and most cultivars are hexaploid (2n = 6x = 90) [18]. Therefore, the construction of high-density genetic maps for *C. oleifera* has always been an enormous challenge. Recently, chromosome-level diploid oil-Camellia genomes (*C. oleifera* var. ‘Nanyongensis’ (CON), *C. lanceoleosa* and *C. chekiangoleosa*, 2n = 2x = 30) have also been reported, and genome sizes of 2.95 Gb, 3.00 Gbs and 2.73 Gb were obtained, respectively [18,19,20]. The diploid “CON” is considered to be the ancestor species of the hexaploid *C. oleifera* [18], and the “CON” genome sequence is advantageous to hexaploid *C. oleifera* breeding. To date, only one genetic map of *C. oleifera* has been constructed, which has poor marker density, with only 300 SNP markers [18]. To date, no research on QTLs associated with *C. oleifera* traits has been published. *C. oleifera* often has larger fruit, a thinner pericarp, and higher oil content in the kernel, allowing for a boost in seed oil yield [18]. However, an understanding of the molecular basis of the regulation of fruit and seed development and oil and fatty acid biosynthesis in *C. oleifera* is limited [21,22,23,24]. Marker development and key gene mining based on a high-density genetic map and fine-scale QTL mapping are necessary for boosting the genetic improvement of oil traits in *C. oleifera*. 

For the construction of the high-density genetic maps, single nucleotide polymorphism (SNP) markers representing the most abundant sources of variation in the genome are being increasingly utilized [25]. In terms of genotyping methods, double digest restriction site-associated DNA sequencing (ddRAD-Seq) is a way for de novo SNP discovery and genotyping in non-model species, even for polyploid plants with high heterozygosity and without reference genomes, that is efficient and cost-saving [26]. In addition, due to this complexity, researchers often use a double pseudo-testcross mapping strategy to construct genetic maps. ddRAD-Seq has already been applied in high-density genetic linkage map construction and QTL mapping in many oil plants, such as peanut [27,28], *Brassica napus* [29], oil palm [30], and sesame [31]. Based on this, nine QTLs for leafspot resistance in peanut were identified and the phenotypic variance explained (PVE) ranged from 11% to 24%; moreover, 30 QTLs for yield-related traits were identified in which three major QTLs showed a PVE of over 10% in sesame. Miao et al. examined the overlapping QTLs of the seed lignin content, cellulose content, hemicellulose content, and oil content in *Brassica napus*, which were found to have a PVE ranging from 9.87 to 48.50% [14]. These results provided enriched QTLs and potential candidates for oil crops yield and quality, as well as new information for understanding the complex genetic mechanism underlying yield formation and oil accumulation.

In this study, we constructed a high-density genetic linkage map using an F_1_ population consisting of 180 progenies and provided high-density molecular markers for genetic breeding. The variances of eleven fruit- and oil-related traits were analyzed based on three years’ worth of data of this F_1_ population. We also performed QTL mapping of these eleven traits. A subset of major QTLs involved in fruit development and oil biosynthesis were obtained. The potential candidate genes in QTL regions were identified based on the collinearity of the linkage map and the *C. oleifera* “CON” genome [18]. This work reveals the pivotal genetic basis underlying fruit development and seed oil biosynthesis and provides insights into genetic improvements in tree breeding.

## 2. Results

### 2.1. Variation and Correlation Analysis of Fruit- and Oil-Related Traits

**Figure 1:**
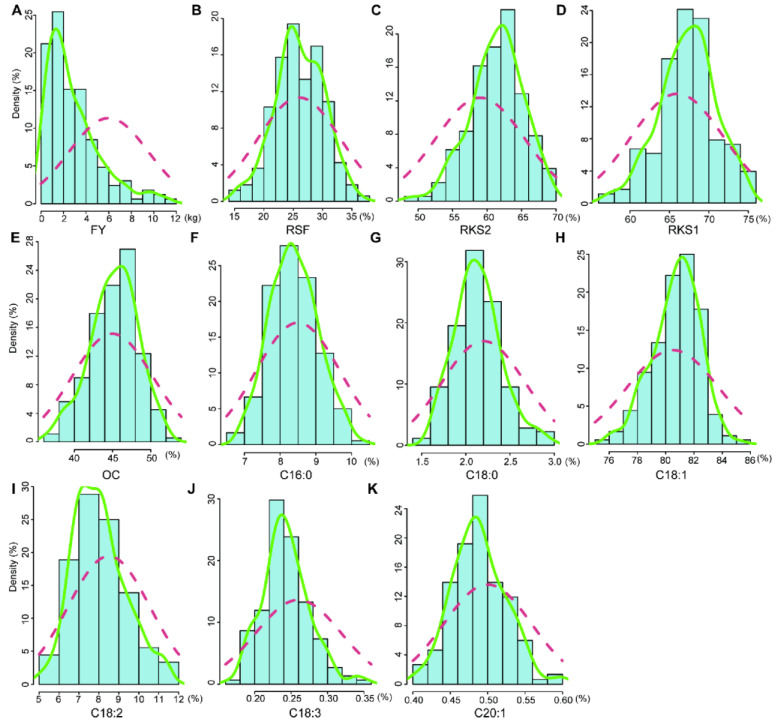
Phenotypic evaluation of eleven traits for the linkage population in 2017. The Y-axis is the percent of plant individuals to the linkage population. The X-axis is the continuous (**A**) fruit yield per plant (FY, kg), (**B**) the ratio of dry seed to fresh fruit (RSF, %), (**C**) the ratio of air-dried kernel to absolute dry seed (RKS2, %), (**D**) the ratio of absolute dry kernel to absolute dry seed (RKS1, %), (**E**) the oil content of absolute dry kernel (OC, %), (**F**) palmitic acid (C16:0, %), (**G**) stearic acid (C18:0, %), (**H**) oleic acid (C18:1, %), (**I**) linoleic acid (C18:2, %), (**J**) linolenic acid (C18:3, %), and (**K**) cis-11-eicosenoic acid (C20:1, %) contents of Camellia oil, respectively. The green line indicates the distribution of plant individuals and the red dashed line indicates the normal distribution curve.

Eleven fruit- and oil-related traits of the mapping population were measured for three consecutive years. As expected, most traits had abundance variance and followed an approximately normal distribution (Figure 1 and Figure S1). These traits were suitable for QTL mapping. For example, the fruit yield per plant (FY) ranged from 0 to 11.34 kg with a mean of 2.62 kg in 2017 and the ratio of dry seed to fresh fruit (RSF) was from 15.43% to 36.36% with a mean value of 26.04%. The oil content of dry kernel (OC) ranged from 16.1% to 52.17% (mean 45.04%). The palmitic acid (C16:0) and oleic acid (C18:1) content in oil, which accounted for over 90% of all fatty acids in Camellia oil, ranged from 6.91% to 9.84% (mean 8.39%) and from 75.20% to 84.12% (mean 80.70%), respectively (Table S1). The variances of eleven traits in 2015 and 2016 resembled the scenario in 2017 (Table S1, Figures S1 and S2).

**Figure 2:**
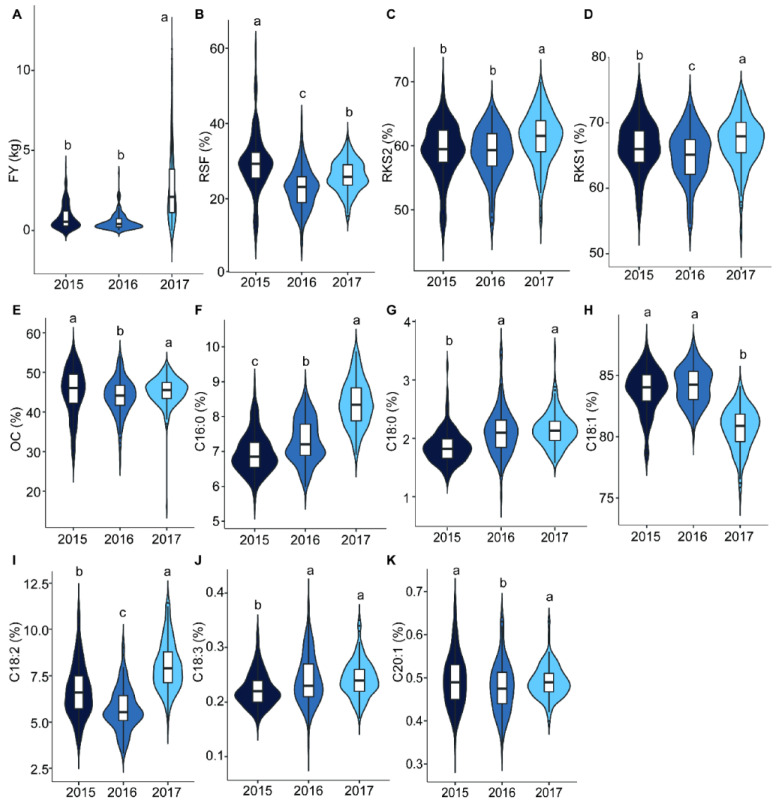
Phenotypic variance of eleven traits among different years. The X-axis represents the years. The Y-axis indicates the (**A**) FY (kg), (**B**) RSF (%), (**C**) RKS2 (%), (**D**) RKS1 (%), (**E**) OC (%), (**F**) C16:0 (%), (**G**) C18:0 (%), (**H**) C18:1 (%), (**I**) C18:2 (%), (**J**) C18:3 (%), and (**K**) C20:1 (%) content of the Camellia oil, respectively. Different lowercase mean the significant differences at *p* < 0.05.

**Figure 3:**
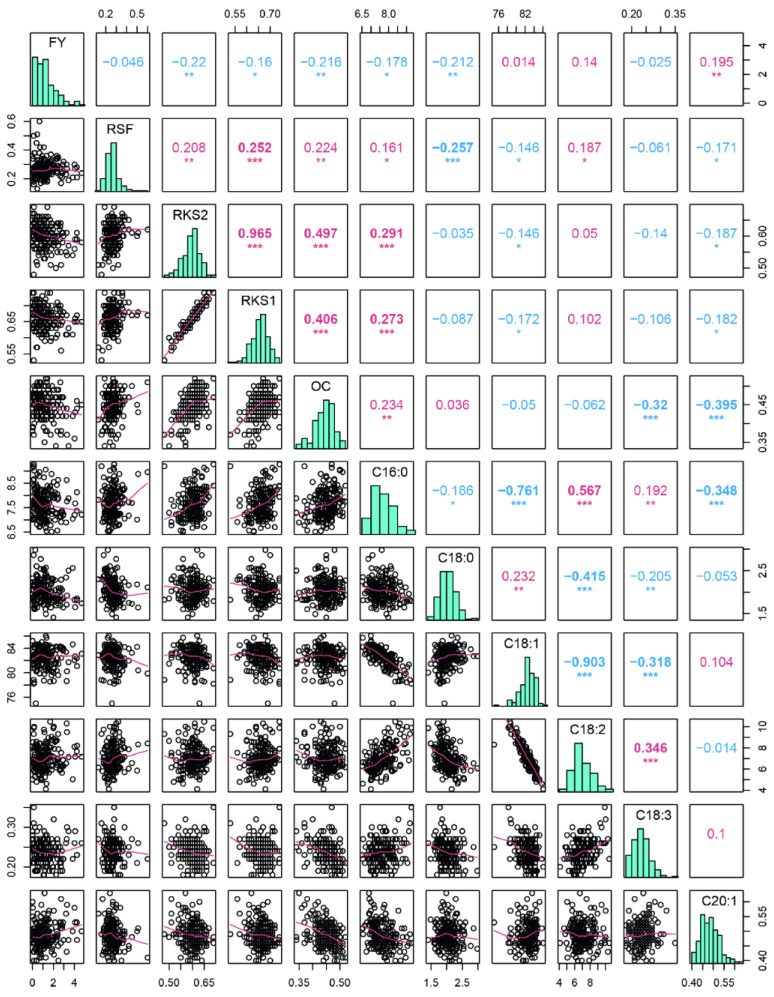
Pearson correlation matrix for eleven fruit- and oil-related traits of the linkage population based on the means of three years. The distribution of the means of eleven traits in three years are shown on the diagonal. To the bottom left are the bivariate scatter plots with best-fit lines displayed. Correlation coefficients are shown above the diagonal. “***”, “**”, and “*” denote significance with *p*-values of 0.001, 0.01, and 0.05, respectively. Red and blue denote positive and negative correlations, respectively.

The analysis of variance (ANOVA) showed that the eleven traits differed significantly among the years (Table 1 and Figure 2). *C. oleifera* fruit yield has the major or minor year phenomenon and it is a major year in 2017. The average FY in 2017 was 2.62 kg, significantly higher than that of the other years. The ratios of dry kernel to dry seed (RKS) and C16:0 and linoleic acid content (C18:2) were significantly higher, and the C18:1 was lower in 2017 than in 2015 and 2016, respectively. A Pearson’s correlation analysis of the eleven traits was performed using the mean values of three years. The fruit- and oil-related traits showed significant correlation in the mapping population (Figure 3). RKS1 and RKS2 were significantly positively correlated (*r* = 0.965, *p* < 0.01), and both were positively correlated with OC with *r* = 0.406 and 0.497 (*p* < 0.01), respectively. In addition, RKS1 and RKS2 were both positively correlated with C16:0 (*p* < 0.01). The RSF was significantly negatively correlated with C18:0 (*p* < 0.01). The six kinds of fatty acid content detected in this study showed significant correlations with each other. According to the correlation, the six fatty acid content traits could be divided into two groups. One group included C16:0, C18:2, and linolenic acid (C18:3), and the other group consisted of stearic acid (C18:0), C18:1, and cis-11-eicosenoic acid (C20:1). Significant positive intra-group pairwise correlations and negative inter-group pairwise correlations were observed (Figure 3). Similar to the previous study results in the association population [6], C18:1 and C18:2 showed significant negative correlations (*r* = 0.903), while C16:0 and C18:2 were positively correlated (*r* = 0.567) in this linkage population (Figure 3).

### 2.2. Analysis of ddRAD Data and SNP Markers

In total, 182 ddRAD libraries (including 180 F_1_ individuals and two parents) were sequenced, which generated approximately 657.84 Gb data [18] with a Q30 of over 94.56%. 4.41 Gb and 5.42 Gb of data were generated for the female and male parents, respectively, with an average 3.60 Gb for every F_1_ individual. The GC contents were ranged from 38.31% to 47.96%, with a mean value of 42.40%. The clean reads were aligned with the reference genome, and over 90% reads could be mapped to the reference genome in 172 samples. The average mapping rate of the reads reached 96.10% for all samples, and the average sequencing depth of the mapped reads reached 10.39X (Table S2).

**Figure 4:**
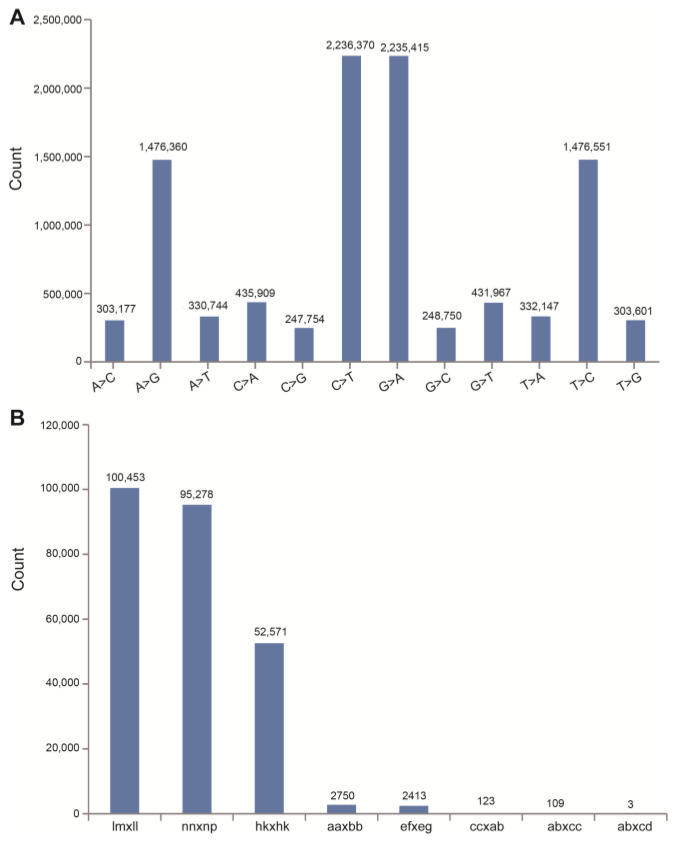
Statistics for variation types (**A**) and segregation patterns (**B**) of SNPs in the linkage population. (**A**): The X-axis indicates the variation types and the Y-axis indicates the SNP numbers for each variation type. (**B**): The X-axis represents the predicted genotype segregation patterns which are labeled by “female genotype × male genotype”. The Y-axis represents the SNP numbers of each segregation pattern. Only the SNPs with the segregation patterns lm × ll, nn × np, hk × hk, and ef × eg were subjected to genetic map construction using the double pseudo-testcross strategy. Lm × ll, nn × np, hk × hk, and ef × eg were matched with heterozygous (female) × homozygous/absent (male), homozygous/absent (female) × heterozygous (male), heterozygous (female) × heterozygous (male), and heterozygous/absent (female) × heterozygous/absent (male) models in the double pseudo-test-cross strategy, respectively. Details of the SNP genotypes of individuals are shown in Table S4.

Next, 1,371,271 and 2,313,900 SNPs were identified in the female and male parents with a heterozygosis rate of 43.20% and 48.35%, respectively, using the *C. oleifera* “CON” genome as a reference [18]. The numbers of SNPs in F_1_ individuals ranged from 240,081 to 2,583,535 with an average of 1,272,218 (Table S3). In all SNPs, the nucleotide transition was significantly more than transversion. The rates of nucleotide transition to transversion (*Ti*/*Tv*) were 3.43 and 3.33 in two parents and from 2.87 to 3.75 in F_1_ individuals, respectively (Table S3). It was found that the base substitution types of C→T and G→A were the most common types, accounting for 22.23% and 22.22% of all SNPs, respectively (Figure 4A). A→G and T→C were the second common types, accounting for 14.68% of all SNPs, respectively. The remaining eight substitution types were relatively few, only accounting for 2.46–4.33% of all SNPs (Figure 4A). The SNP data were further filtered, and a total of 253,700 SNPs were identified with eight kinds of segregation patterns (Figure 4B). In addition, 250,715 SNP markers with the segregation patterns of lm × ll, nn × np, hk × hk, and ef × eg were used for the genetic map construction based on the double pseudo-testcross strategy. The details of all the SNPs used for genetic map construction can be found in *S1 Data* by Lin et al. [18].

### 2.3. Genetic Map Construction and Its Basic Characteristics

**Figure 5:**
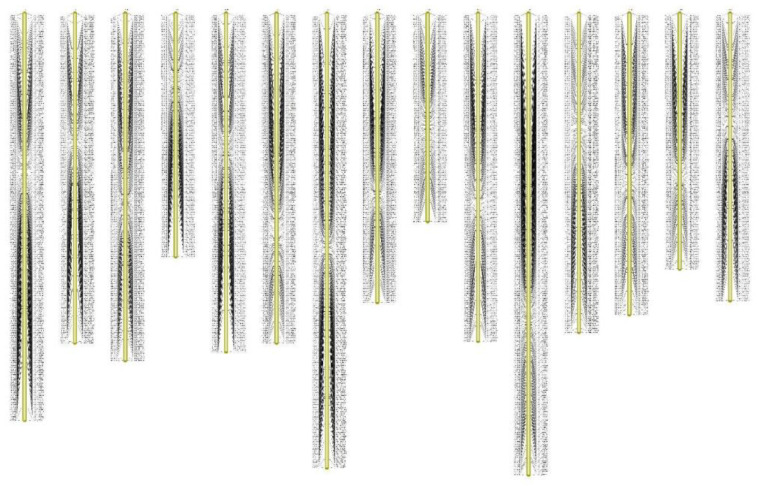
ddRAD based high-density genetic map of 180 full-sibs. The 3327.021 cM map included 2780 SNPs.

**Figure 6:**
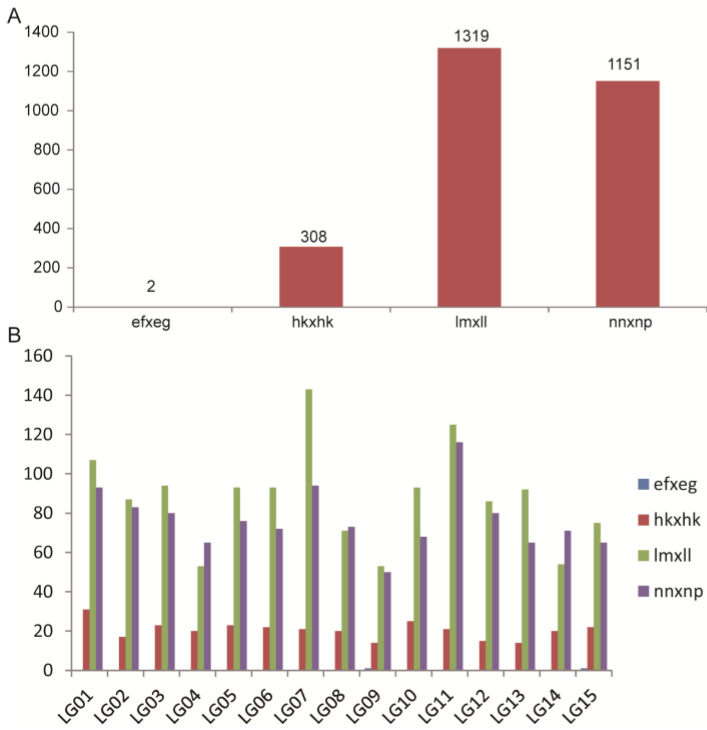
SNP type statistics for the genetic map. (**A**) The SNP numbers of four segregation patterns in the whole genetic map. (**B**) The SNP numbers of four segregation patterns in each linkage group.

The SNPs were further filtered for <70% integrity using a Chi-squared test with a threshold of *p* < 0.01. As a result, a total of 5772 high-quality markers, including 2871 lm × ll, 2371 nn × np, 527 hk × hk, and three ef × eg, were ultimately obtained for map construction (genotype data are listed in Table S4). These SNPs were distributed into 15 chromosomes according to their physical locations on the *C. oleifera* “CON” genome. Then, the marker order and genetic distances of the SNP markers were evaluated. The final genetic map consisted of 15 LGs with 2780 markers and covered a total of 3327.021 cM with an average interlocus distance of 1.20 cM (Figure 5). Information of all the markers on the map can be found in Table S5, including marker IDs, genetic position (cM), chromosomes, and physical location (bp) in the “CON” genome. In all the markers on the map, there were significant differences (*p* < 0.01) among the SNP numbers of different segregation patterns. The lm × ll and nn × np markers were the most common and accounted for 47.45% and 41.40% of all markers, respectively. There were only two ef × eg markers (Figure 6A). The ratio of SNP numbers with different segregation patterns in each LG was analyzed and found to be similar to that for the whole genetic map (Figure 6B).

On average, each LG contained 185 markers that spanned an average length of 221.80 cM (Table 2). The genetic length of 15 LGs ranged from 196.04 cM (LG13) to 319.60 cM (LG07). The LGs were numbered according to the chromosome numbers of the “CON” reference genome. Among the 15 LGs, LG11 was the most saturated, containing 262 markers with an average marker density of 0.86 cM. LG04 contained the largest intervals of 40.67 cM between adjacent markers. The longest linkage group, LG07, harbored 258 markers, covering a length of 319.60 cM with an 1.24 cM average interlocus distance. The shortest linkage group, LG13, contained only 171 markers, spanning a length of 196.04 cM, with an average interlocus distance of 1.15 cM (Table 2).

### 2.4. QTL Analysis of Fruit- and Oil-Related Traits Based on the High-Density Genetic Map

Based on the high-density genetic map and phenotypic data of three consecutive years, the QTLs for eleven fruit- and oil-related traits were identified, and the proportions of the QTLs explaining the phenotypic variance were calculated. In this study, overlapped QTLs or adjacent QTLs with a distance of less than 5 cM were classified into the same loci. Based on this rule, a total of 221 QTLs were identified for the eleven traits of *C. oleifera*, which were distributed among 15 linkage groups, with 10–32 for each phenotypic trait (Table 3 and Table S6). In addition, 670 markers were associated with all the QTLs (Table S6), and 17 major QTLs were found for the eleven traits with a PVE ≥13.0% and LOD ≥4.00.

#### 2.4.1. Fruit-Related Traits

The QTLs for four fruit-related traits, FY, RSF, RKS1, and RKS2, were analyzed. In total, 23 QTLs were identified for FY in all LGs except for LG08, 12, and 14, with an LOD from 3.00 to 4.07 and a PVE from 7.4% to 9.9%, respectively (Table S6). There were 69 markers that were closely linked to these QTLs. 

**Figure 7:**
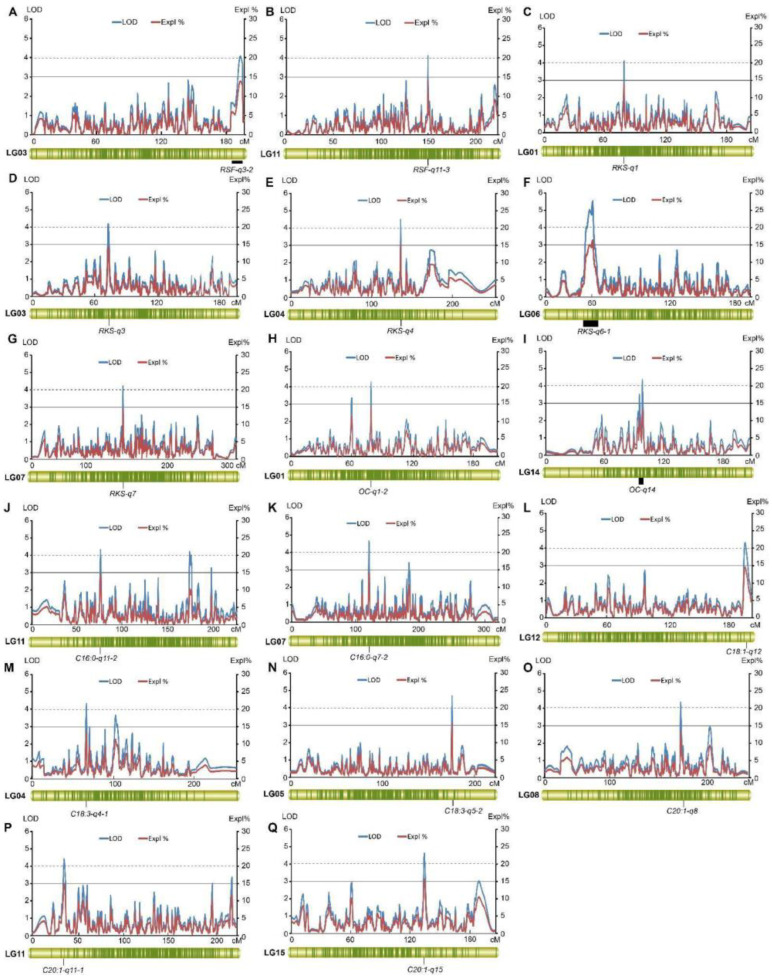
The results of the major QTL test for RSF (**A**,**B**), RKS (**C**–**G**), OC (**H**,**I**), C16:0 (**J**,**K**), C18:1 (**L**), C18:3 (**M**,**N**), and C20:1 (**O**–**Q**).

For the RSF, we found 32 QTLs in LG02 (23.95–24.46 cM, 112.21–113.10 cM), LG03 (131.77–132.34 cM, 184.77–195.40 cM), LG04 (31.57–75.40 cM), LG06 (68.91–70.79 cM, 184.01–186.42 cM), LG07 (119.02–163.38 cM), LG08 (97.76–167.49 cM), LG09 (75.76–89.30 cM, 191.98–211.33 cM), LG10 (63.51–64.59 cM, 138.77–143.93 cM), LG11 (104.21–210.15 cM), LG12 (80.44–81.16 cM), LG13 (54.74–149.15 cM), and LG14 (66.61–119.86 cM), which explained 8.20~14.00% of the observed RSF variation. There were 84 markers closely linked to these QTLs (Table S6). Two major QTLs for RSF were detected in LG03 (184.77–195.40 cM) and LG11 (150.45–151.33 cM), which explained 13.90% and 14.00% of the observed RSF phenotypic variation, and their LODs were 4.07 and 4.1, respectively (Figure 7A,B). Their flanking molecular markers were Chr03_112839138 and Chr11_81012082.

**Figure 8:**
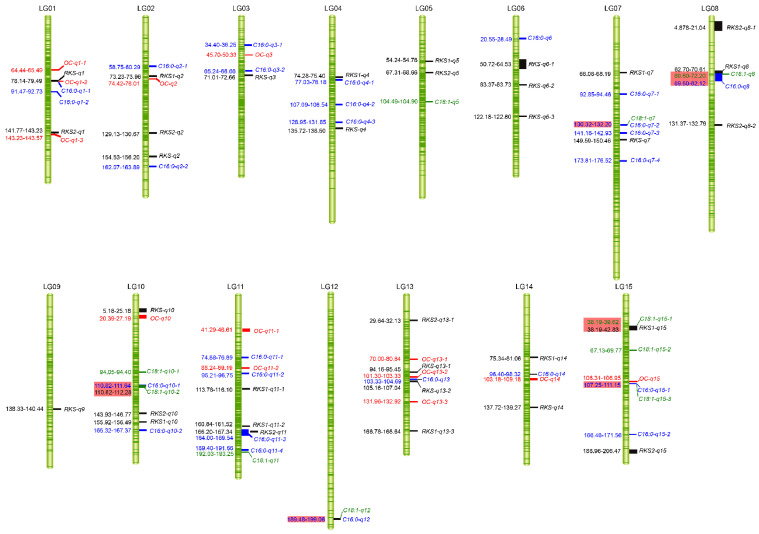
The results of the QTL test for the RKS, OC, C16:0, and C18:1 content. The left of each linkage group shows the location of the QTLs in units of cM. The right shows the QTL name. In this study, the QTLs were named as follows: “target traits (capital letter)” + “−” + “q” + “linkage group code” + “QTL number”. The full name of the QTL is presented in italics. The QTLs for RKS, OC, C16:0, and C18:1 are in black, red, blue, and green, respectively. RKS1 and RKS2 are the percent of absolute dry kernel weight and air-dried kernel weight to the absolute dry seed weight, respectively. RKS indicates the overlapping QTLs for RKS1 and RKS2. The locations in pink rectangles indicate the overlapping QTLs of two traits.

For RKS, a total of 46 QTLs were detected: 24 QTLs for RKS1 and 22 for RKS2. Additionally, 13 QTLs overlapped because of the high correlation of the RKS1 and RKS2 phenotypic data. These QTLs were located in LG01 (78.14–79.49 cM, 141.77–143.23 cM), LG02 (73.23–73.96 cM, 129.13–156.20 cM), LG03 (71.01–72.66 cM), LG04 (74.28–75.40 cM, 135.72–138.50 cM), LG05 (54.24–68.66 cM), LG06 (50.72–83.73 cM, 122.18–122.80 cM), LG07 (66.08–68.19 cM, 149.59–150.46 cM), LG08 (4.88–21.04 cM, 62.70–70.61 cM, 131.37–132.79 cM), LG09 (138.33–140.44 cM), LG10 (5.18–25.18 cM, 143.93–156.49 cM), LG11 (113.78–116.10 cM, 160.84–167.34 cM), LG13 (29.64–32.13 cM, 94.16–107.04 cM, 166.78–168.64 cM), LG14 (75.34–81.06 cM, 137.72–139.27 cM), and LG15 (38.19–42.83 cM, 188.96–206.47 cM) with LODs from 3.00 to 5.57 and the PVEs of 7.50~16.60% (Figure 8 and Table S6). There were 95 markers that were closely linked to these QTLs.

Further, we found five major QTLs for RKS in LG01 (78.14–79.49 cM), LG03 (71.01–72.66 cM), LG04 (135.72–138.50 cM), LG06 (50.72–64.53 cM), and LG07 (149.59–150.46 cM). These QTLs exhibited LODs from 4.13 to 5.57 and explained 13.80–16.60% of the observed RKS phenotypic variation, respectively (Figure 7C–G). Nine flanking SNPs were identified, including Chr01_39071315, Chr03_108338909, Chr04_77743434, Chr06_142599472 (Chr06_112292496, Chr06_88890750, Chr06_25329856 and Chr06_111554923), and Chr07_133837987.

In conclusion, a total of 101 QTLs controlling fruit-related traits and seven major QTLs were detected (Table S6).

#### 2.4.2. Oil-Related Traits

The QTLs for OC and six kinds of fatty acid contents were mined. Thirteen QTLs for OC were detected, of which three were located in LG01 and LG13, with two genetic intervals of 12.65 cM and 63.74 cM in LG01 and 20.46 cM and 28.63 cM in LG13; two in LG11, with a genetic interval of 41.63 cM; and one each in LG02, LG03, LG10, LG14, and LG15 (Figure 8). The LODs of these QTLs were from 3.03 to 4.37, and the PVEs were 7.50–13.30% (Table S6). There were 43 SNP markers closely linked to these QTLs. Two major QTLs for OC located in LG01 (78.141–79.493 cM) and LG14 (103.179–109.183 cM) were found with LODs of 4.28 and 4.37 (Figure 7H,I). These QTLs explained 14.5% and 13.3% of the observed OC variation, respectively. The flanking SNP markers of these two QTLs were Chr01_39071315 and Chr14_62200937 (Chr14_49268251).

Twenty-six QTLs for C16:0 in LG01 (78.035–78.844 cM, 91.466–92.726 cM), LG02 (58.745–60.291 cM, 162.07–163.893 cM), LG03 (34.404–36.251 cM, 65.24–66.66 cM), LG04 (77.03–78.179 cM, 107.093–108.543 cM, 128.952–131.849 cM), LG06 (20.548–28.486 cM), LG07 (92.854–94.457 cM, 130.315–132.196 cM, 141.16–142.925 cM, 173.807–176.519 cM), LG08 (69.597–82.121 cM), LG10 (110.824–111.642 cM, 165.323–167.372 cM), LG11 (74.883–76.89 cM, 95.209–96.752 cM, 164.001–169.537 cM, 189.399–191.663 cM), LG12 (189.484–199.058 cM), LG13 (103.325–104.692 cM), LG14 (96.397–98.324 cM), and LG15 (107.254–111.151 cM, 166.456–171.564 cM) were identified with a PVE of C16:0 from 7.90% to 14.60% (Figure 8 and Table S6). There were 87 markers closely linked to these QTLs. Two major QTLs located in LG07 and LG11 were identified with LODs of 4.31 and 4.58 and PVEs of 14.6% and 13.9%, respectively (Figure 7J,K). Chr07_96522586 and Chr11_64280487 were the flanking SNPs of these two QTLs.

Ten QTLs for C18:1 were identified in LG05 (104.487–104.896 cM), LG07 (130.315–132.196 cM), LG08 (69.597–72.195 cM), LG10 (94.045–94.404 cM, 110.824–112.279 cM), LG11 (192.03–193.251 cM), LG12 (189.484–199.058 cM), and LG15 (38.187–39.621 cM, 67.133–69.77 cM, 107.254–111.151 cM) (Figure 8). The LODs ranged from 3.00 to 4.30, and these QTLs explained 10.40–14.50% of the C18:1 content variation (Table S6). There were 32 markers that were closely linked to these QTLs. Only one major QTL located in LG12 was identified with an LOD of 4.30, and it explained 14.50% of the observed C18:1 variation (Figure 7L). The flanking SNP of this QTL was Chr12_74070604.

Moreover, 13, 14, 28, and 16 QTLs for C18:0, C18:2, C18:3, and C20:1 were also detected in the whole genetic map, respectively (Table S6). The markers linked to these QTLs ranged from 41 to 94 for these four oil-related traits. The LODs were from 3.01 to 4.57, and these QTLs could explain 7.70–15.4% of the corresponding phenotypic variations. A total of five major QTLs had LOD values over 4.0 and PVE ≥ 13.0%, which included two for C18:3 and three for C20:1, respectively (Figure 7M–Q). The major QTL for C20:1 located in LG15 had an LOD of 4.57 and explained 15.4% of the observed C20:1 variation, which was the QTL with the highest LOD in this group of QTLs.

In conclusion, a total of 120 QTLs controlling oil-related traits and 10 major QTLs were detected. Of all the QTLs, nine could be detected simultaneously using at least two years’ worth of trait data, and 48 markers were associated with these OTLs (Table 4 and Table S6). The nine QTLs were composed of two for RKS2, RKS1, and C18:2, and one for C18:3, C16:0, and C18:0, and with a 7.5–16.6% PVE for the corresponding traits. The PVEs showed significant differences among the three years, and the PVEs detected based on 2017 phenotypic data were significantly lower than those based on the 2015 and 2016 data. The lengths of these QTLs ranged from 1.159 cM (C18:2-q5-1) to 13.81 cM (RKS2-q6-1), and 25 SNPs were located in these QTL regions. Of these 25 markers, four loci had three genotypes in the linkage population and the parents were both heterozygotes. The other loci had two genotypes in the linkage population; one of parents was a heterozygote and the other was a homozygote. The mean values of the phenotype differed somewhat between the different genotype groups (Table 4). Furthermore, the dominant and additive effects of the four SNPs, with three genotypes in the linkage population, were calculated (Table 5). It was shown that the modes of gene action were different based on the data from different years. The marker–trait pair Chr06_25329856-RKS2 acted with the mode of under- or overdominance (|d/a| > 1.25) in 2015 and 2016 and with the mode of dominance (0.50 < |d/a| < 1.25) in 2017, which resembled the scenario for the Chr05_93231328-C18:2 pair. Chr06_25329856-RKS1 acted with the mode of dominance in 2015, under- or overdominance in 2016, and codominance (additive) gene action (|d/a| < 0.50) in 2017, respectively. The Chr02_98558620-C18:0 pair acted with the mode of under- or overdominance (|d/a| = 2.33) in 2015 and dominance in 2016 and 2017 (|d/a| = 0.86, 0.50), respectively (Table 5). 

### 2.5. Identification of Fruit- and Oil-Related Candidate Genes and Potential Regulatory Network

Based on the collinearity relationship between the genetic linkage map and the *C. oleifera* “CON” reference genome, 89 and 113 potential candidate genes in *C. oleifera* were identified as being involved in fruit development and oil biosynthesis, respectively (Table S7). Some noteworthy potential fruit-related candidates were identified in the regions of FY-, RSF- and RKS-QTLs. For instance, *Chr11_gene_1254.12* and *Chr15_gene_52.4* corresponding to *CoERF053* and *CoERF061*, the transcription factors that regulate vegetable oil biosynthesis [32,33,34,35], were located in the confidence interval (CI) region of *RKS1-q11-2* and *FY-q15-2* QTLs, respectively. *Chr05_gene_78.2* corresponding to *CoSAUR67*, the transcription factor that regulates fruit development and maturation [36], was located in the CI region of *FY-q5-1*. *Chr10_gene_428*, which is homologous to PLC2 (phosphoinositide phospholipase C2), an enzyme involved in TAG assembly in the endoplasmic reticulum, was mapped to the region of *RKS1-q10-2*. 

A number of potential oil-related candidates were identified in the regions of OC-, C16:0-, C18:0-, C18:1-, C18:2-, C18:3-, and C20:1-QTLs. *Chr15_gene_748.45*, which was homologous to WRI1 (ethylene-responsive transcription factor), a transcriptional activator involved in the activation of a subset of sugar-responsive genes and the control of carbon flow from sucrose import to oil accumulation in developing seeds [37], was mapped to the overlapping region of four QTLs: *C16:0-q15-2*, *C18:0-q15*, *C18:1-q15-3,* and *C18:3-q15-1*. *Chr02_gene_151.2,* corresponding to *CoARF2B*, was located in the CI region of *C18:0-q2*. *Chr06_gene_1017.3,* corresponding to *CoARF23*, was located in the overlapping region of *C16:0-q6* and *C18:2-q6*. *CoARF2B* and *CoARF23* were the transcription factors belonging to the *ARF* family and were integral to the regulation of fruit set [38]. *Chr02_gene_806.11,* corresponding to *CoSAD*, a desaturase involved in the de novo biosynthesis process of free fatty acids in plastid, was located in the region of *C18:2-q2-2*. *Chr03_gene_1405.12,* corresponding to *CoDOF3.6*, the transcription factor that may augment the lipid content of plant seeds by upregulating genes associated with the biosynthesis of fatty acids [39], was located in the CI region of *C16:0-q3-2*.

**Figure 9:**
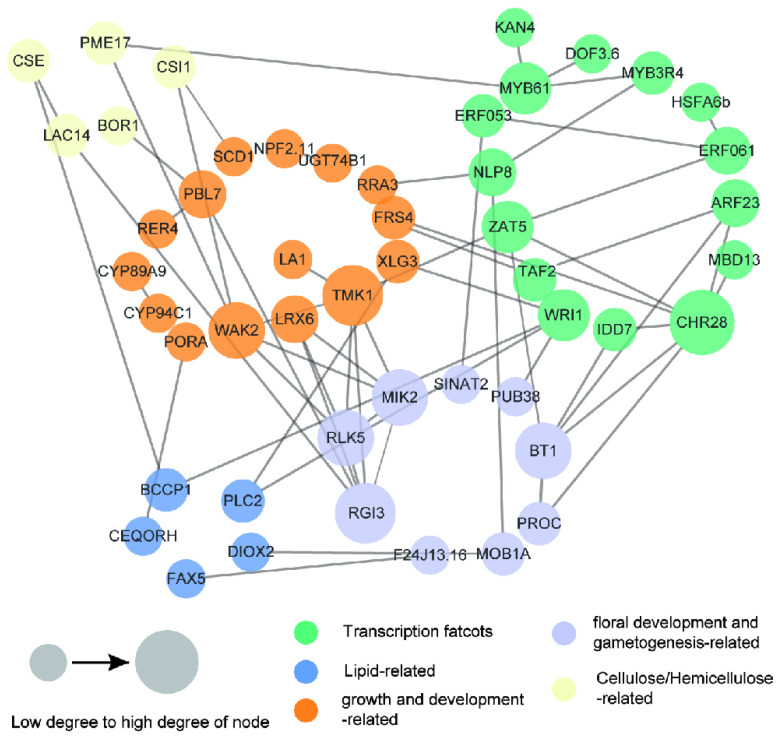
Interaction network of potential candidate genes for fruit- and oil-related traits. Network visualization of the interaction of the 49 candidate genes and regulatory factors constructed using String software and observed using Cytoscape_V3.7.1. Genes are presented as nodes, and gene interactions are presented as edges.

To further dissect the fruit development and oil accumulation mechanism in *C. oleifera*, an interaction network of the potential candidate genes in QTL regions was constructed based on *Arabidopsis thaliana* orthologs (Figure 9). The whole network, including 34 enzymes and 15 transcription factors, was incorporated into 49 nodes and 114 edges. A number of important genes and regulatory factors involved in growth and development; floral development and gametogenesis; and the accumulation of oil, cellulose, hemicellulose, and lignin in seeds were observed, such as *WAK2*, *BT1*, *BCCP1*, *CSE*, *LAC14*, and *WRI1* (Figure 9). *WAK2* might interact with *MIK2* or *RLK5* to regulate the pollen tube perception of the female signal and floral organ abscission. *WAK2* might also interact with *CSI1* to function in primary cell wall biosynthesis and cellulose microfibril organization. Four transcription factors, *ZAT5*, *IDD7*, *ARF23*, and *CHR28*, might associate with *BT1* to participate in the regulation of bloom and gametophyte development. *WRI1* might interact with *BCCP1* to control the carbon flow from sucrose import to oil accumulation in the developing seeds. *CSE* might interact with *LAC14* to function in the biosynthesis and degradation of lignin. *CSE* might also interact with *BCCP1* to regulate oil accumulation.

## 3. Discussion

### 3.1. Mapping Population and Phenotypic Variation

The selection of mapping population is important for constructing a high-density map and further QTL analyses. Recombinant inbred line (RIL) or F_2_ populations are commonly used to construct genetic maps for most crops and vegetables [28,31,40,41,42]. The challenges in hybrid population creation and conservation have hindered the progress of tree genetic population construction. F_1_ populations are usually used to construct genetic maps for trees that have high heterozygosity, a long generation cycle, and from which it is difficult to obtain pure lines [43,44,45,46,47]. In this study, we used the F_1_ population, derived from a cross between CL53 and CL81, for constructing a high-density genetic map and subsequent QTL mapping of fruit- and oil-related traits. In previous studies, this population was used to construct the first linkage map of *C. oleifera* containing 300 SNP markers [18]. Fruit yield, oil content in the kernel, and the fatty acid component were the important determinants of the economic and meal value of *C. oleifera*. The improvement in oil yield and quality, including fruit yield, the rate of seed to fruit, kernel to seed, oil content in the kernel, and main fatty acid component, can enhance the economic and meal value [6,18,48]. Variances in the yield- and oil-related traits were abundant in the various populations of *C. oleifera* [6,18,48]. In this F_1_ population, FY, RSF, RKS, OC, and six kinds of fatty acid contents all showed abundant phenotypic variation among individuals and years (Figure 1 and Figure 2). RKS was positively correlated with OC, C16:0, and C18:2 and negatively correlated with C18:1. These results were identical to previous reports noting that C18:2 had a positive correlation with C16:0 and a negative correlation with C18:1 in the *C. oleifera* natural population [6,18,49]. It is noteworthy that C16:0 was highly positively correlated with C18:2 (*r* = 0.567, *p* < 0.01) (Figure 3).

### 3.2. Density and Accuracy of the C. oleifera Genetic Map

The construction of genetic linkage maps and the identification of important trait-related QTLs will facilitate future genetic and breeding studies in *C. oleifera*. The number and distribution of markers in the genome determine the uniformity and coverage of the markers in the genetic map [9,50]. It has been demonstrated that increasing marker density can improve the resolution of genetic maps and QTL in a given mapping population [51,52]. However, the existence of linkage disequilibrium (LD) implies a limitation on the effectiveness of increasing marker density to improve the resolution of genetic maps. A suitable marker density in the genetic map could be theoretically saturated [53]. To date, only one *C. oleifera* genetic map has been constructed, but the marker density is low (300 markers), with an average interlocus distance of 6.46 cM [18]. No high-density genetic map for any oil-tea Camellia species had been generated, thus preventing the use of QTLs from *C. oleifera* in breeding programs. ddRAD is a rapid, efficient, and cost-effective strategy for SNP development, genetic linkage map construction, marker-based complex trait selection, and draft genome assembly in many species with or without reference genomes [25,28,46,54,55,56,57,58]. In the present study, we generated 657.84 Gb of raw data using the ddRAD-seq technique, with an average of 3.60 Gb for each F_1_ individual. Here, 1,371,271 and 2,313,900 SNPs were detected from the two parents. The genetic map was divided into 15 LGs and comprised 2780 SNP markers, which covered 3327.021 cM with an average marker interval of 1.20 cM. This represented the most saturated linkage map of *C. oleifera* to date. The high-density SNP-based linkage map provides detailed information on genomic structure and is a valuable resource for fine-scale QTL mapping, MAS, and genomic studies, which should contribute to the genetic improvement of *C. oleifera*.

### 3.3. QTL Analysis of Economic Traits in C. oleifera

Fruit yield, oil content in the kernel, and fatty acid component are complex quantitative traits controlled by multiple genes and environmental factors. We have some understanding of the effect of individual locus on the economic traits of *C. oleifera* and their position in the genome [6,18]. However, these loci discovered in *C. oleifera* natural populations explained only 1.87–17.93% of the variation in the OC and fatty acid component [6,18]. QTL mapping is an efficient tool to screen candidate genes that control quantitative traits. However, few reports have identified the loci that control *C. oleifera* traits using QTL analysis. In our QTL mapping analysis, we developed 221 QTLs from eleven economic traits, which explained 7.40–16.60% of the phenotypic variation, respectively (Table 3 and Table S6). We also found that 101 genomic loci distributed on all 15 LGs were associated with fruit-related traits and 120 loci were associated with oil-related traits (Table 3 and Table S6). These regions may contain the primary candidate genes that control the variation in fruit- and oil-related traits.

Some chromosome segments may be associated with different traits for different growth periods and environmental factors, and QTLs can be detected throughout various stages of plant development [59,60,61]. Some QTLs are conditional and are only found in specific growth stages or environments [9,62,63,64]. The *RKS1-q6-1* located in the interval 54.07–64.53 cM on LG06 appeared to be unconditional and was detected in all three years’ measurements in this study (Table S6). The other eight QTLs were reproduced in two years, including *RKS2-q4*, *RKS2-q6-1*, *RKS1-q4-2*, *C18:3-q11-2*, *C18:2-q5-1*, *C18:2-q11-1*, *C18:0-q2,* and *C16:0-q11-3*. The remaining 95.93% of QTLs were only detected in one year (Table S6). In this study, the 26 QTLs associated with C16:0 content were distributed on all LGs except LG05 and LG09 (Figure 8 and Table S6). Ten QTLs for C18:1 content were detected on the seven LGs, including LG05, LG07, LG08, LG10, LG11, LG12, and LG15 (Figure 8 and Table S6). It is worth noting that five out of the ten QTLs affecting C18:1 content, located in the interval 130.32–132.20 cM on LG07, 69.60–72.20 cM on LG08, 110.82–112.28 cM on LG10, 189.48–119.06 cM on LG12, and 107.25–111.15 cM on LG15, were wholly or partially overlapped with the QTLs for C16:0 content (Figure 8). A possible explanation for the many overlapping QTLs for different traits is that these economic traits are regulated by the interactions of many genes that may act in the same metabolic pathway.

### 3.4. Identification and Analysis of the Candidate Genes

A number of potential genes involved in oil metabolism in *C. oleifera* have been reported. Lin et al. [18] identified 21 high-confidence candidate genes, including 14 genes associated with oil accumulation and seven involved in fatty acid content. These candidate genes are located on almost all chromosomes, except Chr01, Chr04, Chr09, and Chr14 in *C. oleifera*. It is worth noting that these candidate genes include not only genes involved in the lipid metabolism pathway, such as malonyl-CoA:ACP transacylase (FabD), ketoacyl-ACP synthase II and III (KASII and KASIII), stearoyl-ACP desaturase (SAD), fatty acid desaturase 2 (fad2), Oleosin 3, and PLC6, but also phytohormone-related transcription factors, for example, auxin-responsive protein (IAA14 and IAA26), auxin response factor (ARF11), and ethylene response factor (ERF4 and ERF5) [6,18,49,65,66,67,68,69]. In the present study, a total of 202 candidate genes involved in fruit development and lipid metabolism were identified (Table S7). Similarly, some functional genes related to fatty acid biosynthesis and some phytohormone-related transcription factors were detected. For example, the transcription factors *CoERF053* and *CoERF061*, which regulate the biosynthesis of vegetable oil [32,33,34,35], were identified as being associated with the RKS and FY in *C. oleifera* for the first time. *CoSAUR67*, which governs fruit development and maturation [36], was identified in the FY QTLs in this study. The interaction network analysis showed that a number of important candidate genes and regulatory factors interacted with each other and might play a potential role in fruit development and lipid biosynthesis in *C. oleifera*.

## 4. Materials and Methods

### 4.1. Plant Materials

*C. oleifera* clone CL53 is a widely cultivated variety in China, with high fruit yield and moderate OC (mean 36.70%) and C18:1 (mean 79.46%). Clone CL81 normally has a lower fruit yield but higher OC (mean 44.53%) and C18:1 (mean 82.02%). A controlled crossing was performed using “CL81” and “CL53” as male and female parents in the autumn of 2009 at Dongfanghong Forest Farm of Zhejiang Province, Jinhua City, China. At the end of October 2010, 220 seeds were harvested and sown in the middle of March 2011. In 2013, two-year-old seedlings were raised from nursery and planted with a row spacing of 3 m, a plant spacing of 2 m, and a randomized complete block design with three replications. We selected two parents and 180 healthy F_1_ individuals from this population for genotyping and mapping analyses.

### 4.2. Phenotypic Data Collection

Oil yield and quality, which depend on fatty acid composition, are the important breeding aims of *C. oleifera*. In this study, phenotypic data related to oil yield and quality were collected when the seeds of the F_1_ population and two parents matured in the autumns of 2015, 2016, and 2017, respectively. A total of eleven key traits were measured using the following methods: (1) FY (kg), the weight of all fresh fruits in each individual; (2) RSF (%), the average percent of absolute dry seed weight to fresh fruit weight in 30 fruits for one tree; (3) RKS (%), the average percent of absolute dry kernel weight (RKS1) or air-dried kernel weight (RKS2) to absolute dry seed weight in 30 fruits for one tree; (4) OC (%), the average percent of oil weight to absolute dry kernel weight in 30 fruits for one tree. The Soxtec extraction method was performed to measure the OC as described [19]; (5) C16:0 (%), C18:0 (%), C18:1 (%), C18:2 (%), C18:3 (%), and C20:1 (%) contents of *C. oleifera* oil, the average weight percent of every kind of fatty acid to all fatty acids in 30 fruits for one tree. The total lipid was extracted from the kernels using petroleum ether, and six kinds of fatty acid components were quantified using gas chromatography according to the previous study [6,70,71].

The normal fitting of the phenotypic data, the ANOVA, and the Pearson’s correlation coefficients (*r*) for the eleven traits were calculated using the Data Processing System (DPS v14.50) [72] and the pairs function in R (https://www.r-project.org/) (accessed on 1 January 2023).

### 4.3. DNA Extraction

Tender, healthy leaves were harvested from the 180 F_1_ individuals and their parents, and DNA was extracted using the TaKaRa MiniBEST Plant Genomic DNA Extraction Kit (TaKaRa, Dalian, China) according to the user manual. The DNA was quantified with an ND-2000 spectrophotometer (NanoDrop, Wilmington, DE, USA) and by electrophoresis in 1.2% agarose gels.

### 4.4. ddRAD Libraries Sequencing and SNPs Identification

The ddRAD library preparation and sequencing, SNP identification, and genotyping were performed by Lin et al. [18]. After filtering, only SNPs with the segregation patterns of lm × ll, nn × np, hk × hk, and ef × eg were used for the genetic map construction based on the double pseudo-testcross strategy [73]. These four kinds of markers were matched with heterozygous (female) × homozygous/absent (male), homozygous/absent (female) × heterozygous (male), heterozygous (female) × heterozygous (male), and heterozygous/absent (female) × heterozygous/absent (male) models in the double pseudo-testcross strategy, respectively.

### 4.5. Genetic Map Construction

First, the SNP markers used for genetic map construction were further filtered. The significantly distorted segregation markers with *p*-values less than 0.01 in the chi-square test and a marker integrity < 30% were excluded. Lin et al. constructed the first genetic map with a poor marker density using the “CON” genome as a reference genome [18]. In this study, the genetic map was constructed by a strategy without a reference genome. The marker order and genetic distances in each LG were calculated using JoinMap4.1 [74]. The regression mapping algorithm and Kosambi’s mapping function were used for map construction with the parameters of recombination frequencies ≤ 0.4, logarithm of odds (LODs) ≥ 3.0 and Jump = 5 [75]. A graphic genetic map was generated using a custom perl script (https://github.com/Niuyongchao/Fish_linkage_map, accessed on 20 December 2022).

### 4.6. QTL Mapping and Genetic Mode Analysis of SNPs 

The inclusive composite interval mapping (ICIM) program of QTL Icimapping v4.2 (https://isbreeding.caas.cn/rj/qtllcmapping/294445.htm, accessed on 4 January 2023) [76] was used to detect the QTLs for oil-related traits using the *C. oleifera* F_1_ population. Two steps were included in the ICIM. In the first step, stepwise regression was applied to identify the most significant regression variables. In the second step, a one-dimensional scanning or interval mapping was conducted for detecting additive (and dominance) QTLs and a two-dimensional scanning was conducted for detecting digenic epistasis. A window size of 10 cM and a walk speed of 1 cM were chosen for ICIM analysis. The significance levels of the input and output regression equations (PINs) were 0.002, and the LOD threshold was 3.0. QTL scanning was performed for each trait using three consecutive years of phenotypic data, respectively. For each QTL, the position corresponding to the maximum LOD and the part of the phenotypic variation it explained was estimated. The QTLs with PVE ≥ 13.0% and LOD ≥ 4.00 were considered major QTLs.

The ratios of dominance (*d*) to additive (*a*) effect were calculated for the significant markers to quantify the mode of gene action according to previous research [77,78].

### 4.7. Identification of Fruit- and Oil-Related Candidate Genes and Potential Regulatory Networks

The QTLs for fruit- and oil-related traits obtained in this research were aligned into the physical map of the “CON” reference genome [18]. The closely linked markers of QTLs were used to locate the genomic position. The genes within QTL regions that had SNPs in introns, exons, or within 1 kb up- or downstream were regarded as potential candidate genes. Functional predictions of candidate genes were performed according to the annotation of the “CON” genome. In addition, The interaction networks were analyzed and constructed using String software (http://string-db.org/, accessed on 14 December 2023) and visualized by Cytoscape V3.7.1 [79]. The degree centrality were calculated by a network analyzer and mapped as the node size. All the interactions, clusters, and analyses were based on *A. thaliana* orthologs.

## 5. Conclusions

The most saturated high-density SNP genetic map for *C. oleifera* to date was constructed based on an F_1_ population with 180 progenies. The polymorphic markers were developed using the method of ddRAD-Seq. A total of 657.84 Gb of raw data were generated, with an average of 3.60 Gb for each F_1_ individual. The genetic map included 2780 markers on 15 LGs and covered 3327.021 cM with an average marker interval of 1.20 cM. Based on this high-density genetic map and the phenotypic data from three consecutive years, a QTL analysis of eleven fruit- and oil-related traits was performed. A total of 221 QTLs were detected with PVEs from 7.40% to 16.60% in which 17 major QTLs had PVEs > 13% and LODs ≥ 4.0. The flanking SNPs adjacent to the major QTLs were identified and could serve as potential molecular markers for the breeding of *C. oleifera*. A total of 202 high-confidence candidate genes involved in fruit development and lipid metabolism were detected, including the transcription factors *CoSAUR67*, *CoERF053*, *CoARF23*, *CoDOF3.6*, and *CoWRI1* and the function genes *CoPLC2* and *CoSAD*. These results will not only provide a platform for gene/QTL fine mapping, map-based gene isolation, and molecular breeding in *C. oleifera* but also provide a reference to help position sequence scaffolds on the physical map and assist in the process of assembling the *C. oleifera* genome sequence.

## Figures and Tables

**Table 1:** Analysis of variance for eleven fruit- and oil-related traits in the linkage population of *C. oleifera*.

Sources of Variation		Year	Genotypes	Error
*df*		2	179	358
FY	MS	269.4273	2.8638	1.8835
	*F* value	143.049 **	1.521 **	
RSF	MS	3488.9907	72.3878	37.9708
	*F* value	91.886 **	1.906 **	
RKS2	MS	291.5984	27.5792	6.5039
	*F* value	44.834 **	4.24 **	
RKS1	MS	268.7622	26.0167	5.7166
	*F* value	47.015 **	4.551 **	
OC	MS	75.0375	27.4846	15.3854
	*F* value	4.877 **	1.786 **	
C16:0	MS	102.5323	0.5838	0.1932
	*F* value	530.75 **	3.022 **	
C18:0	MS	4.6355	0.1403	0.0551
	*F* value	84.199 **	2.549 **	
C18:1	MS	657.5575	4.0707	1.4652
	*F* value	448.777 **	2.778 **	
C18:2	MS	245.2011	2.6365	0.8135
	*F* value	301.403 **	3.241 **	
C18:3	MS	0.0264	0.0018	0.0008
	*F* value	33.577 **	2.266 **	
C20:1	MS	0.0143	0.004	0.0015
	*F* value	9.765 **	2.754 **	

MS, Mean squares for phenotypes; **, *p* < 0.01.

**Table 2:** Genetic linkage group statistics for the genetic map.

LG ID	Total Markers	Total Distance (cM)	Average Distance (cM)	Gaps > 5 cM(%)	Max Gap (cM)
LG01	231	203.79	0.89	1.74	20.62
LG02	187	220.89	1.19	5.91	14.28
LG03	197	196.62	1.00	2.04	10.63
LG04	138	253.02	1.85	5.84	40.67
LG05	192	222.04	1.16	4.71	26.96
LG06	187	196.19	1.05	2.69	12.75
LG07	258	319.60	1.24	4.28	19.77
LG08	164	262.68	1.61	7.98	16.16
LG09	118	211.33	1.81	5.98	19.35
LG10	186	205.40	1.11	2.70	15.22
LG11	262	224.30	0.86	2.30	22.44
LG12	181	201.07	1.12	2.22	10.37
LG13	171	196.04	1.15	2.35	19.84
LG14	145	207.60	1.44	5.56	15.29
LG15	163	206.47	1.27	5.56	17.51
Total	2780	3327.02	1.20	3.88	40.67
Average	185	415.88	1.20	3.88	---

**Table 3:** The statistics of QTLs for fruit- and oil-related traits in *C. oleifera*.

Traits	Year	Number of QTLs	Linkage Group	PVE ^a^ (%)	LOD Value
FY	2015	10	1, 2, 5, 6, 10, 13	7.4–9.4	3.0–3.87
	2016	5	3, 5, 7, 9	7.4–9.0	3.02–3.7
	2017	8	3, 4, 7, 11, 13, 15	7.6–9.9	3.07–4.07
RSF	2015	14	2, 4, 6, 7, 9, 10, 11, 14	9.3–11.4	3.0–3.72
	2016	8	2, 3, 6, 7, 8, 11	10.5–14.0	3.01–4.1
	2017	10	3, 9, 10, 12, 13, 14	8.2–11.2	3.06–4.25
RKS2	2015	5	1, 6, 8, 10, 11	9.8–16.6	3.17–5.57
	2016	14	1, 2, 3, 4, 6, 7, 8, 9, 13, 14, 15	10.4–15.1	3.0–4.48
	2017	5	4, 5, 6, 10, 13	7.5–9.0	3.02–3.66
RKS1	2015	5	2, 6, 10, 11	9.6–14.3	3.09–4.71
	2016	14	1, 2, 3, 4, 6, 7, 9, 13, 14, 15	10.6–14.5	3.08–4.27
	2017	8	4, 5, 6, 7, 8, 10, 14	7.5–10.0	3.0–4.08
OC	2015	6	3, 13, 14, 16	9.4–13.3	3.03–4.37
	2016	4	1, 10, 11	11.5–14.5	3.36–4.28
	2017	3	1, 2, 11	7.5–8.3	3.03–3.35
C16:0	2015	9	1, 2, 7, 8, 11, 13, 15	9.5–13.9	3.07–4.58
	2016	11	1, 3, 4, 7, 10, 11, 12, 14, 15	10.4–14.6	3.02–4.31
	2017	7	3, 6, 7, 10, 11	7.9–11.9	3.17–4.89
C18:0	2015	5	2, 5, 7, 13	9.7–12.5	3.13–4.09
	2016	5	3, 4, 12, 15	10.4–12.6	3.01–3.67
	2017	4	2, 9, 13, 14	7.7–8.8	3.09–3.56
C18:1	2015	4	5, 7, 8, 15	10.1–12.2	3.26–3.99
	2016	6	10, 11, 12, 15	10.4–14.5	3.0–4.3
	2017	0			
C18:2	2015	5	3, 5, 7, 11, 15	9.6–10.8	3.07–3.5
	2016	7	2, 6, 10, 11, 12, 13	10.5–13.4	3.05–3.95
	2017	4	5, 10, 11	7.5–9.8	3.0–3.99
C18:3	2015	14	1, 3, 4, 8, 9, 10, 11, 14, 15	9.4–13.0	3.02–4.26
	2016	9	3, 4, 5, 7, 11, 12, 13	10.5–15.3	3.05–4.54
	2017	6	2, 3, 5, 11, 13, 15	8.0–9.7	3.2–3.93
C20:1	2015	5	1, 7, 8, 10, 13	9.5–10.2	3.05–3.31
	2016	9	4, 7, 9, 10, 11, 13, 15	10.5–15.4	3.02–4.57
	2017	2	1, 11	7.7–8.1	3.09–3.27

^a^ Percentage of phenotypic variation explained by each QTL.

**Table 4:** Summary of QTLs identified in at least two years and the traits in the full-sib population of “ChangLin No. 53” (CL53) × “ChangLin No. 81” (CL81).

Trait	QTL	Start	End	Length (cM)	Year	LOD	PVE (%)	Marker	Cross Type	CL53 (♀)	CL81 (♂)	Genotype	Individual Numbers	Phenotype Means(%)		
														2015	2016	2017
RKS2	*RKS2-q4*	135.721	138.501	2.78	2016	4.48	15.1	Chr04_77743434	lm × ll	GT	GG	GG	101		59.70	62.05
					2017	3.01	7.5					GT	76		58.24	60.33
												-	3		57.78	61.94
	*RKS2-q6-1*	50.723	64.534	13.811	2015	5.57	16.6	Chr06_142599472	lm × ll	GA	GG	GG	97	59.25		61.07
					2017	3.66	9.0					GA	81	59.12		61.56
												-	1	62.77		62.97
								Chr06_112292496	lm × ll	GA	GG	GG	97	59.31		61.22
												GA	80	59.33		61.58
												-	3	54.52		56.69
								Chr06_88890750	lm × ll	CT	CC	CC	100	59.57		61.35
												CT	77	58.85		61.29
												-	3	56.83		60.14
								Chr06_25329856	hk × hk	AG	AG	AA	35	57.95		61.19
												AG	96	59.58		61.24
												GG	30	59.01		61.50
												-	19	59.93		61.57
								Chr06_111554923	lm × ll	GA	GG	GG	91	59.44		61.36
												GA	86	58.87		61.20
												-	3	61.85		62.65
RSK1	*RKS1-q4-2*	135.721	138.501	2.78	2016	4.27	14.5	Chr04_77743434	lm × ll	GT	GG	GG	101		64.78	67.21
					2017	3.28	8.1					GT	76		64.65	66.97
												-	3		63.40	67.17
	*RKS1-q6-1*	54.072	64.534	10.462	2015	4.71	14.3	Chr06_142599472	lm × ll	GA	GG	GG	97	65.67	64.48	
					2016	3.37	11.6					GA	81	66.71	65.26	
												-	1	69.27	68.23	
								Chr06_112292496	lm × ll	GA	GG	GG	97	66.14	64.75	
												GA	80	66.23	64.72	
												-	3	62.76	61.66	
								Chr06_88890750	lm × ll	CT	CC	CC	100	66.31	65.23	
												CT	77	65.99	64.04	
												-	3	62.91	61.79	
								Chr06_25329856	hk × hk	AG	AG	AA	35	65.24	63.62	
												AG	96	66.25	65.11	
												GG	30	66.19	64.90	
												-	19	66.77	64.22	
C18:3	*C18:3-q11-2*	84.52	85.695	1.175	2015	3.16	9.8	Chr11_27083024	nn × np	CC	CT	CC	86	22.24		24.45
					2017	3.93	9.7					CT	92	22.23		24.53
												-	1	31.47		33.96
C18:2	*C18:2-q11-1*	162.869	166.196	3.327	2015	3.07	9.6	Chr11_114235680	nn × np	CC	CT	CC	98	6.54		7.96
					2017	3.02	7.5					CT	80	6.90		8.05
												-	2	6.23		9.21
								Chr11_65073730	lm × ll	AG	AA	AA	78	6.78		8.08
												AG	98	6.59		7.91
												-	4	8.16		9.35
	*C18:2-q5-1*	104.487	105.646	1.159	2015	3.5	10.8	Chr05_107820202	lm × ll	AG	GG	GG	95	6.84		8.22
					2017	3.0	7.5					AG	79	6.55		7.76
												-	4	7.00		7.62
								Chr05_93231328	hk × hk	TC	TC	TT	56	6.75		7.88
												TC	67	6.87		8.13
												CC	32	6.37		8.15
												-	25	6.58		7.84
C16:0	*C16:0-q11-3*	164.001	169.537	5.536	2015	3.73	11.5	Chr11_39972487	lm × ll	AG	AA	AA	84	6.87		8.37
					2017	4.2	10.3					AG	94	7.02		8.39
												-	2	6.68		8.10
								Chr11_39972501	lm × ll	AG	AA	AA	83	6.85		8.35
												AG	95	7.03		8.41
												-	2	6.68		8.10
								Chr11_39972457	lm × ll	CA	CC	CC	82	6.85		8.35
												CA	96	7.03		8.40
												-	2	6.68		8.10
								Chr11_128763300	nn × np	TT	TA	TT	98	6.88		8.33
												TA	79	7.04		8.44
												-	3	6.47		8.37
								Chr11_10782110	nn × np	GG	GA	GG	78	6.95		8.38
												GA	100	6.95		8.38
												-	2	6.34		8.36
								Chr11_64280835	nn × np	CC	CT	CC	99	6.96		8.38
												CT	78	6.93		8.37
												-	3	6.68		8.46
								Chr11_146396217	lm × ll	GA	GG	GG	79	6.91		8.37
												GA	100	6.97		8.38
												-	1	6.84		8.44
C18:0	*C18:0-q2*	78.596	82.114	3.518	2015	3.13	9.7	Chr02_116850822	lm × ll	AG	GG	GG	99	1.85		2.13
					2017	3.44	8.5					GA	76	1.87		2.17
												-	5	1.75		1.94
								Chr02_98558620	hk × hk	AG	AG	GG	41	1.79		2.11
												GA	86	1.89		2.14
												AA	39	1.85		2.15
												-	14	1.81		2.21

**Table 5:** List of marker effects for QTLs of traits.

Trait	SNP	2*a* ^1^			*d* ^2^			*d/a*			Frequency ^3^		*a* ^4^		
		2015	2016	2017	2015	2016	2017	2015	2016	2017			2015	2016	2017
RKS2	Chr06_25329856	1.06	0.93	0.31	1.10	0.95	−0.11	2.08	2.05	−0.68	0.48	(G)	−0.76	−0.51	0.04
RKS1	Chr06_25329856	0.95	1.28	0.34	0.53	0.85	0.04	1.13	1.33	0.24	0.48	(G)	−0.42	−0.46	−0.07
C18:2	Chr05_93231328	0.38	0.16	0.27	0.31	0.37	0.12	1.63	4.62	0.85	0.42	(C)	−0.11	−0.17	−0.02
C18:0	Chr02_98558620	0.06	0.14	0.04	0.07	−0.06	0.01	2.33	−0.86	0.50	0.49	(A)	−0.03	0.04	−0.01

^1^ 2*a*, calculated as the difference between the phenotypic means observed within each homozygous class (2*a* = |*G_BB_* − *G_bb_*|, where *G_ij_* is the trait mean in the *ij*th genotypic class). ^2^ *d*, calculated as the difference between the phenotypic mean observed within the heterozygous class and the average phenotypic mean across both homozygous classes [*d* = *G_Bb_* − 0.5(*G_BB_* + *G_bb_*), where *G_ij_* is the trait mean in the *ij*th genotypic class]. ^3^ Allele frequency of the minor allele. Single nucleotide polymorphism (SNP) alleles corresponding to the frequency listed are given in the parentheses. ^4^ The additive effect was calculated as *a* = *p_B_*(*G_BB_*) + *p_b_* (*G_bb_*) − *G*, where *G* is the overall trait mean, *G_ij_* is the trait mean in the *ij*th genotypic class, and *p_i_* is the frequency of the *i*th marker allele. These values were always calculated with respect to the minor allele.

## Data Availability

Data is contained within the article and Supplementary Material.

## Supplementary Materials

The supporting information can be downloaded at https://www.mdpi.com/article/10.3390/ijms25168840/s1.
