# Supplementary figures and images for: High-Density Genetic Map Construction and Quantitative Trait Locus Analysis of Fruit- and Oil-Related Traits in Camellia oleifera Based on Double Digest Restriction Site-Associated DNA Sequencing
Source: Int J Mol Sci. 2024 Aug 14;25(16):8840. doi: 10.3390/ijms25168840 (PMC11354348; doi:10.3390/ijms25168840)

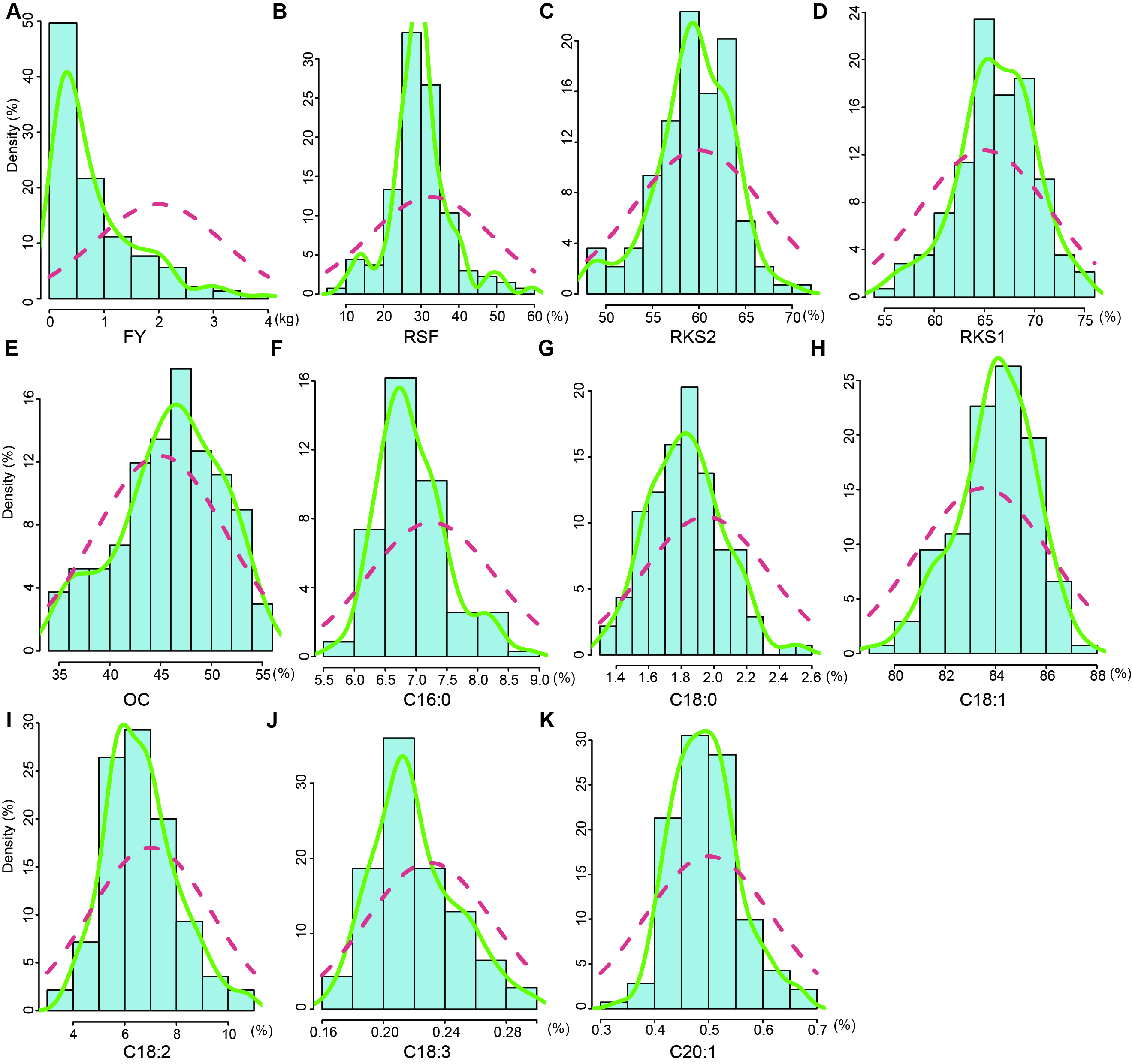

Supplement: Supplementary file 1 [file ijms-25-08840-s001.zip › Figure S1 phenotypic evaluation of eleven traits for linkage population in 2015.tif]

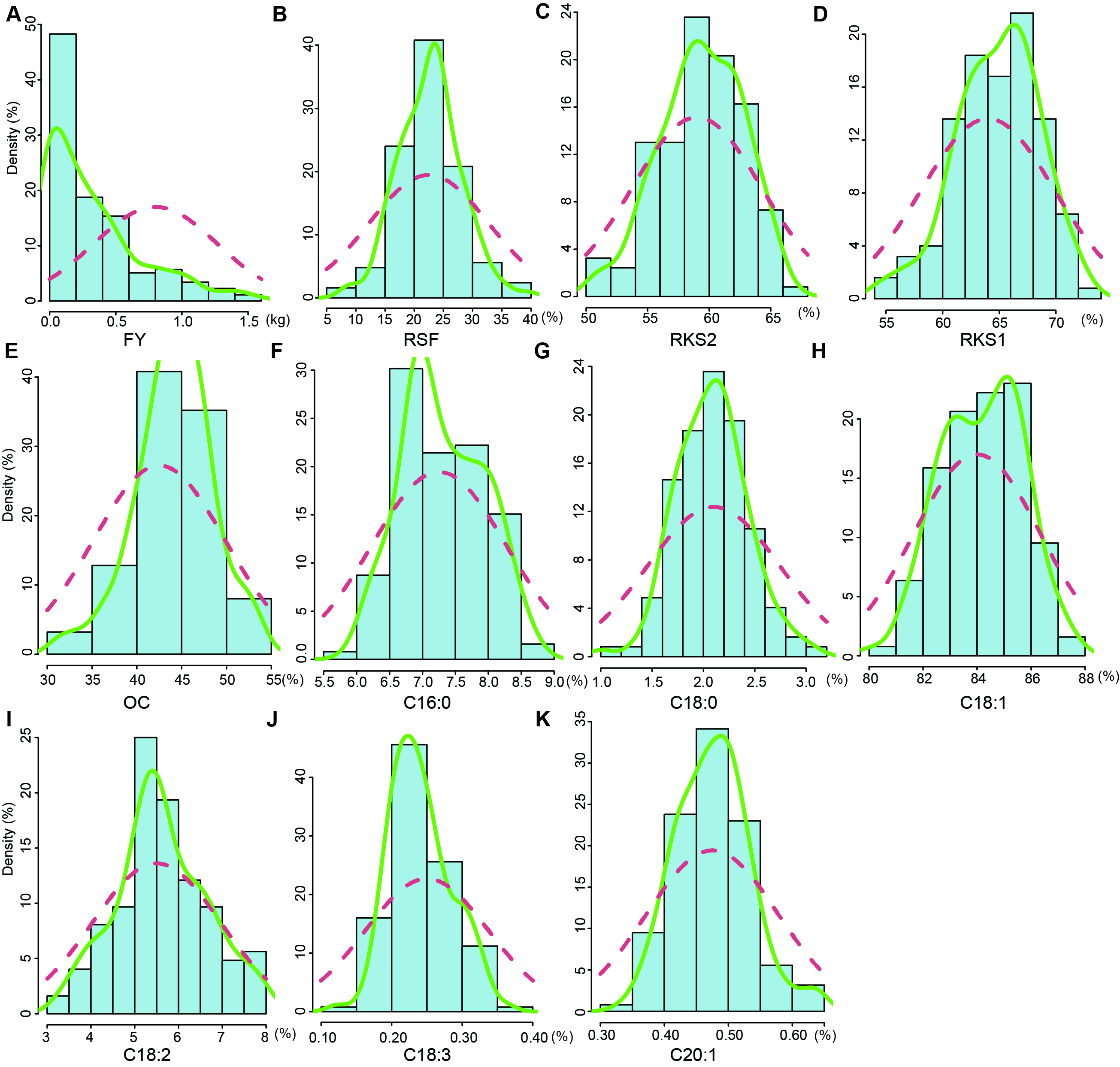

Supplement: Supplementary file 1 [file ijms-25-08840-s001.zip › Figure S2 phenotypic evaluation of eleven traits for linkage population in 2016.tif]
